# A Post‐Traumatic Osteoarthritic Model of Hip Following Fracture of Acetabulum in Rabbit: A Preliminary Study by Macroscopic and Radiographic Assessment

**DOI:** 10.1111/os.12882

**Published:** 2021-01-04

**Authors:** Yanjin Li, Ruibing Feng, Ximing Liu, Guodong Wang, Wei Wang, Qilin Lu, Wei Huang, Haiyang Wu, Xianhua Cai

**Affiliations:** ^1^ College of Acupuncture and Orthopedics Hubei University of Chinese Medicine Wuhan China; ^2^ Department of Orthopaedic Surgery PLA Middle Military Command General Hospital Wuhan China; ^3^ Department of Orthopedics Wuhan Hospital of Traditional Chinese Medicine Wuhan China; ^4^ Department of Orthopedics Hubei Province hospital of Traditional Chinese Medicine Wuhan China; ^5^ Department of Spine Surgery Hubei 672 Orthopaedics Hospital of Integrated Chinese & Western Medicine Wuhan China; ^6^ Department of Spine Surgery Jingmen NO.2 People's Hospital Jingmen China

**Keywords:** Acetabulum, Hip joint, Intra‐articular fractures, Osteoarthritis, Rabbits

## Abstract

**Objective:**

To develop a post‐traumatic osteoarthritic model of hip following fracture of acetabulum in rabbit for revealing biochemical mechanism of post‐traumatic osteoarthritis.

**Methods:**

A total of 36 mature male New Zealand white rabbits were equally divided into sham group (n = 12), non‐ORIF group (n = 12), and open reduction and internal fixation (ORIF) group (n = 12). Except for the sham group, rabbits had survival surgeries to create acetabular fractures of dorsal wall for simulating dashboard impaction mechanism. The ORIF group received open reduction and internal fixation, while fractures in the non‐ORIF group were left as displaced but transverse fracture and dislocation was reduced. Besides intraoperative appearance and postoperative recovery, macroscopic and radiographic characteristics of the hips were recorded and assessed by a radiographic scoring scale at 3 weeks, 6 weeks, and 6 months, respectively.

**Results:**

Out of 24 modeled acetabula, 21 (87.5%) were pure dorsal wall fractures as proposed and the remaining three were associated fractures (dorsal wall plus transverse fracture) accompanied by dorsal dislocation or not. All hips were stable, and no sciatic nerve injury was observed. One rabbit in the ORIF group died of deep infection 4 days after surgery. Rabbits in the sham and ORIF groups returned to normal gait in 2 weeks, but animals in the non‐ORIF group suffered from limping and restricted movement. As the time progressed, the hips in the non‐ORIF group experienced progressive and severe degeneration which exhibited dramatically malformed and hypertrophic joints at 6 months, but the ORIF group maintained much better morphological structure. Corresponding to morphological changes, the average radiographic scores of the non‐ORIF group increased from 1.25 at 3 weeks to 2.75 at 6 months and showed statistically significant difference when compared to the sham group at all three time points (*P* = 0.011, 0.011, 0.015, respectively, <0.0167). Although the scores of the ORIF group showed apparent improvements (increased from 0.67 at 3 weeks to 2.00 at 6 months), there was no significant difference between the two modeled groups at all three time points.

**Conclusion:**

The fracture model with high consistency and reproducibility showed progressive post‐traumatic osteoarthritic changes which could be improved by open reduction and internal fixation surgery and provided an alternative selection for investigating potential pathogenesis and pathology of post‐traumatic osteoarthritis following fracture of acetabulum.

## Graphical abstract

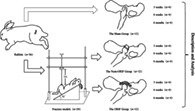

Experimental flowchart. Thirty‐six rabbits were equally divided into three groups. The sham group (n = 12) underwent a sham surgical procedure, while the other two groups underwent modeled procedures, which attempted to simulate dashboard impaction mechanism and create an intra‐articular dorsal wall fracture, with (the ORIF group) or without internal fixation (the non‐ORIF group).

## Introduction

Even as early as 1964, Letounel and Judet first recommended open reduction and internal fixation (ORIF) surgery for acute acetabular fractures which were incongruent or unstable in order to get a better long‐time result^1^. However, the incidence of postoperative complications, mainly comprised of post‐traumatic osteoarthritis (PTOA), avascular necrosis of the femoral head (AVN), and heterotopic ossification (HO), were remained at a high level during the past decades, even though anatomical reconstruction could be fulfilled^2^, ^3^, ^4^, ^5^.

As the most common complication and the main cause of conversion to total hip arthroplasty (THA) following the fracture of acetabulum^6^, PTOA is caused by multifactorial reasons and would result in chronic pain and severe disability. Notwithstanding, the incident ratio of PTOA has declined with accumulated experience of specialists^4^, ^7^, there are as many as 8.5%–15% of patients who had a THA in 2–3 years after preliminary ORIF surgery^5^, ^8^.

The pathogenesis of PTOA is still unclear. Despite cadaveric specimen studies and finite element analysis revealing some biomechanical characteristics of the acetabular fractures^9^, ^10^, ^11^, ^12^, ^13^, ^14^, ^15^, progression toward successful prevention and treatment of PTOA is impeded by the lack of available preclinical animal models, which is indispensable for revealing biochemical mechanism and evaluating the efficacy of therapeutic treatments.

For comprehensive research, a reproducible animal model with consistent fracture pattern and cartilage trauma is prerequisite but also very difficult. During the past decades, most animal models simulated intra‐articular fractures by osteotomies without impaction of cartilage^16^, ^17^, while some others induced cartilage impaction without fracture^18^, ^19^. However, both fracture creation and cartilage impaction are inseparable elements for a high‐energy intra‐articular trauma such as acetabular fracture, in which blunt trauma to cartilage happens simultaneously with fracture. In 2004, Olson *et al*. reported an *in vivo* Nubian goat model for simulating human posterior wall fracture of acetabulum^20^. This model showed a more realistic mechanism involving the two important elements mentioned above. Although there was a significant degeneration in modeled hips at 90 days, this model has not been widely applied yet, which may be related to its some disadvantages such as high cost and long degenerative course. Since 2016, a consistent and reproducible acetabular fracture was successfully developed in a rabbit model by the author's research team^21^. In comparison with Nubian goat model and other large animal models, rabbit models reveal some advantages such as low cost, good adaptability, short growth cycle and disease course, all of which are beneficial to carry out a large sample study.

The goals of the present study were: (i) to develop a post‐traumatic osteoarthritic model of hip following fracture of acetabulum in rabbit for revealing biochemical mechanism of PTOA; (ii) to explore the macroscopic and radiographic changes of the fractured hips in different groups as time progresses; and (iii) to preliminarily assess the effect of open reduction and internal fixation surgery after dorsal wall fracture of acetabulum.

## Methods and Materials

### *Animal Model*

Thirty‐six unselected skeletally mature male New Zealand white rabbits (3.6 ± 0.5 kg, 10–12 months of age) were purchased from WQJX Bio‐technology Co. Ltd. (License No. SCXK20160011, Wuhan, China). Rabbits were individually housed in single cages in a 12/12‐h light/dark cycle, fed with standard laboratory rabbit chow and tap water ad libitum. All procedures performed in the present study involving animals were conducted in accordance with the recommendations of Guide for the Care and Use of Laboratory Animals of the National Institutes of Health and approved by the Ethics Committee of the General Hospital of Central Theater Command (No: 2017019). Radiographs of the pelvis and femur were made to verify closure of the growth plate and no limb abnormality while normal gait was examined. After 2 weeks of acclimation, rabbits were equally divided into the sham group (n = 12), the non‐ORIF group (n = 12), and the ORIF group (n = 12) in accordance with the random number table.

### *Surgery Process*

**Fig 1:**
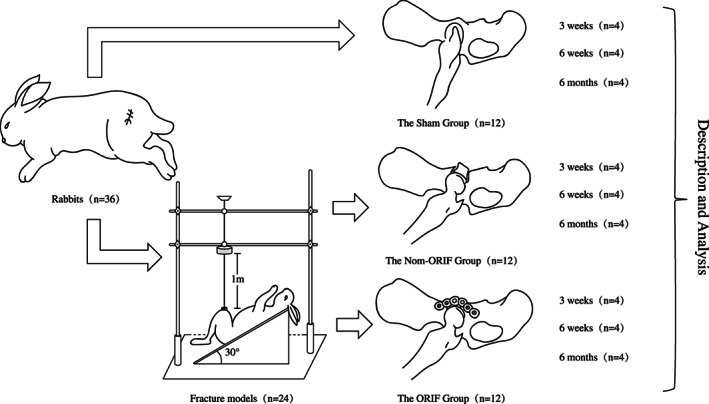
Experimental flow chart. Thirty‐six skeletally mature New Zealand white rabbits were equally divided into three groups. The sham group (n = 12) underwent a sham surgical procedure, while the other two groups underwent modeled procedures, which attempted to simulate dashboard impaction mechanism and create an intra‐articular dorsal wall fracture, with (the ORIF group, n = 12) or without internal fixation (the non‐ORIF group, n = 12). Besides intraoperative appearance and postoperative recovery, macroscopic and radiographic characteristics of the hips were recorded and assessed by a semi‐quantitative scoring system at 3 weeks, 6 weeks, and 6 months, respectively.

Rabbits in non‐ORIF and ORIF groups had survival surgeries under aseptic condition to create acetabular fractures of dorsal wall, simulating dashboard impaction mechanism. No more modeling procedure was made after joint exposure for the sham group (Fig. 1). All surgeries were conducted by the same two surgeons.

For each rabbit, general anesthesia was induced through intravenous injection of pentobarbital sodium (30 mg/kg). After shaving the hair and preparing skin of the left haunch, rabbit was positioned in right lateral recumbency on an operating table, with the left hip uppermost.

The left hip was exposed through a posterior approach. A 5 cm curved incision was made centered on the vertex of greater trochanter, the fascia between the superficial gluteal muscles was incised and deep muscle was split by blunt dissection and retracted to expose the retroacetabular surface. For a few cases, partial tenotomy of the short external rotators, which inserted to the medial surface of greater trochanter, should be carried out to get sufficient field as needed.

**Fig 2:**
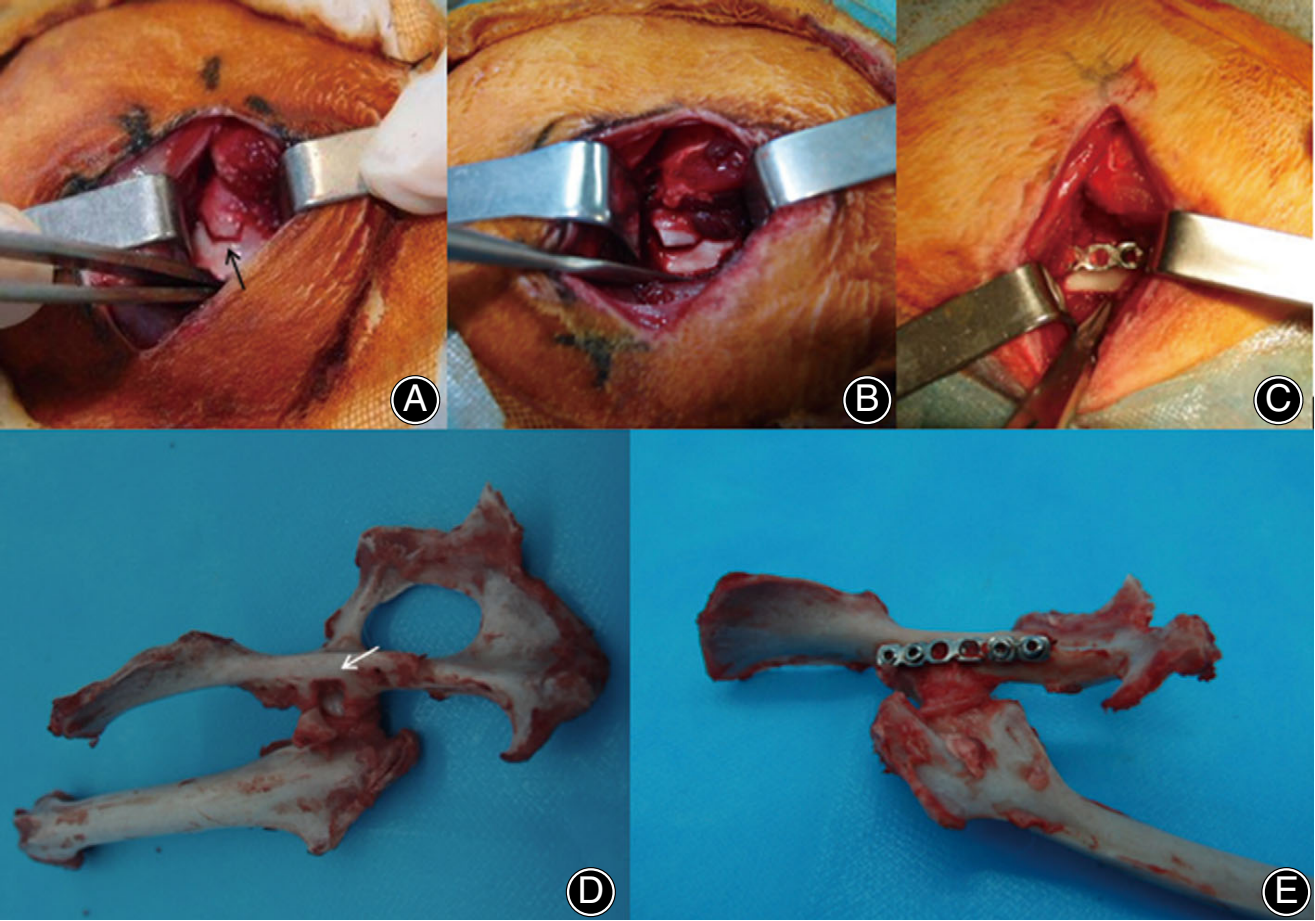
Surgical technique: (A–C) intra‐operative view: (A) the trapezoid fragment (black arrow) on the retroacetabular surface. (B) The fragment was displaced and rose up after impaction. (C) The fragment was reduced and fixed with plate and screws. (D–E) Unilateral hip joint specimen: (D) A hip specimen with a trapezoid bony defect (white arrow) after reversing the fragment. (E) A hip specimen after internal fixation.

A 0.8 mm Kirschner wire drill was used to outline a proposed trapezoid fracture fragment (Fig. 2). The width of the fragment (approximately 3–4 mm according to the individual size of acetabulum) was no more than half of the retroacetabular surface. The cranial part of the outline originated from the joint edge of the epiphyseal line of ischium (located in the central dorsal acetabulum) and was obliquely oriented to the medial line, which was approximately 4 mm long and parallel to the medial edge of ischium, then obliquely turn to the caudal edge of the acetabulum. As a result, a span of approximately 60° along the acetabular rim was included in the bone mass. Subsequently, all of the intermittent drilling holes were meticulously connected with a 3 mm osteotome. It was worth nothing that the drilling and connecting only just passed through the outer cortex but not the articular cartilage at this time and capsular attachment to the fragment should be carefully preserved. The wound was rinsed with saline and covered by an aseptic dressing.

The rabbit was fixed in supine position on a customized worktop with an oblique plane of 30°. The left hip was flexed at approximately 120° and the ipsilateral knee was kept at natural flexion with a plastic brace, so that the axis of the femoral shaft was perpendicular to the center of the proposed dorsal fragment of acetabulum as well as the ground. According to Judet's research on the posterior wall fracture mechanism^1^, the femoral shaft was placed in 0° of abduction in the frontal plane and natural position with internal and external rotation.

**Fig 3:**
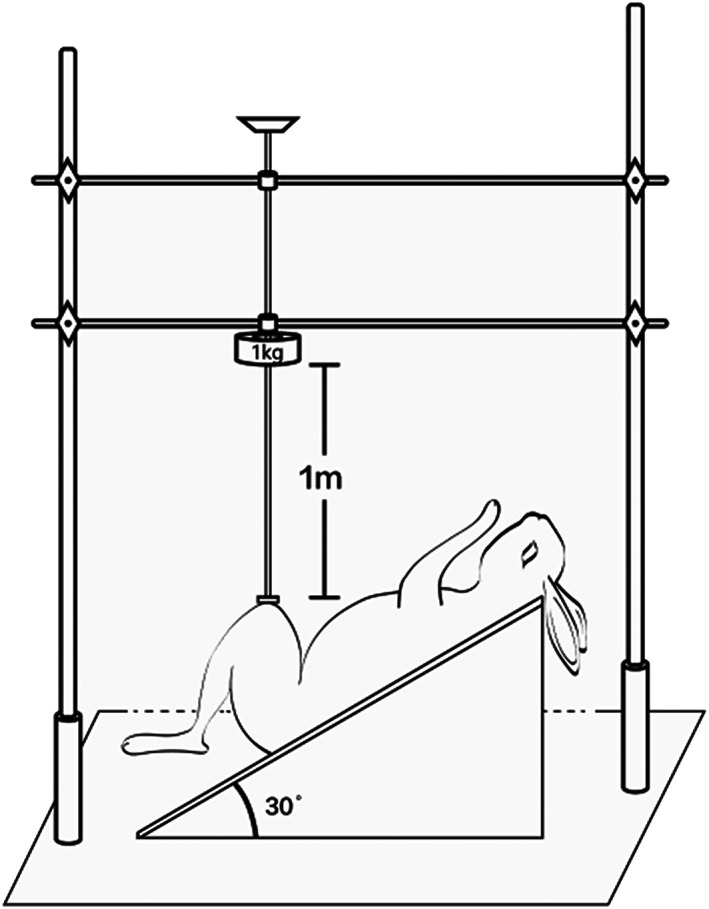
Schematic diagram of drop‐tower impaction for modeling. While an assistant kept the operated limb in the right position, a one kilogram of gravity‐accelerated mass slid down from 1 meter height along the slide‐rod and impacted the left knee.

A customized drop‐tower impacting configuration was applied. The left knee was tightly enfolded by a customized plastic brace. The slide‐rod was kept in line with the femoral shaft as well as perpendicular to the floor. A rubber pad which fitted perfectly with the surface of the plastic brace was fixed between the end of the slide‐rod and the brace surface to ensure a uniform impaction. While an assistant kept the operated limb at right position, a one kilogram of gravity‐accelerated mass slid down from 1 meter height along the slide‐rod and impacted the left knee (Fig. 3).

After impaction, the animal was returned to the operating table and the left hip was inspected. For the ORIF group, the displaced fragment and other debris or dorsal dislocation, if existed, were reduced under direct vision and fixed with a six‐hole, 1.5 mm reconstruction plate system. What was noteworthy was that the plate must be contoured elaboratively to provide a matched buttress for the dorsal wall fragment, which would be kept at anatomic reduction and stable during postoperative daily activity. The plate was secured with two 1.5‐mm bicortical screws above and below the fragment and care was taken to ensure all of the screws were extra‐articular. Moreover, the sciatic nerve, which was no far away from caudal acetabulum, should be carefully protected during operation. The left hip was gently moved in full range of motion (ROM) and it was ensured that there were no abnormal sounds, which was correlated with intra‐articular penetration of screws. For the non‐ORIF group, the fragments were left as displaced, but transverse fracture or dislocation, if they existed, should be reduced to recover their natural position. The stability of the femoral head and the fractures were also inspected. The wound was rinsed with saline and the fascia was closed with single‐knot stitches followed by a continuous suture for skin. Postoperative radiographs were used to assess the models and quality of reduction.

### *Postoperative Care*

After recovering in a warm room, the rabbits were sent back to their individual cages and immediately allowed full weight‐bearing postoperatively. Cefazolin sodium (im, 50 mg/kg/day) was administered as prophylactic antibiotics preoperatively and for the initial 3 days after surgery. Buprenorphine (im, 0.05 mg/kg) was administered twice daily for 5 days as postoperative analgesia.

### *Imaging, Harvesting, and Photographing*

Except for the ORIF group at 3 weeks, in which three rabbits were euthanized since one rabbit died of infection, every four animals for each other group were euthanized at 3, 6 weeks and 6 months, respectively, by overdose of pentobarbital sodium (iv, ≥100 mg/kg).

After removing the hardware, anteroposterior digital radiographs of the pelvis were taken, then left proximal femur and acetabulum of each rabbit were harvested and surrounding soft tissue was meticulously removed. Immediately after taking photographs using a digital camera (XZ‐1, OLYMPUS), the specimens were fixed in neutral buffered formalin.

### *Outcome Measures*

#### *Macroscopic Recording*

According to the photographs of harvested joints, morphological characteristics such as cartilage degeneration and remodeling of acetabulum or femoral head were recorded and preliminarily compared between different groups at different time points.

#### *Radiographic Scoring*

Based on the features of hip joint radiographs, a semi‐quantitative scoring system (The Radiographic OA Scoring System, Table 1), which had been validated in clinical and epidemiological studies^22^, ^23^, was used to assess the presence and severity of post‐traumatic osteoarthritis. A healthy hip joint was scored 0. As disease progressed, score 1 represented mild PTOA and score 2 and 3 indicated moderate and marked PTOA, respectively. Larger numbers indicated increased degrees of PTOA severity. Two senior specialists, who were blinded to the grouping, separately scored the radiographs of each rabbit. When inconsistencies appeared and agreement could not be reached, a third specialist was invited to make a final decision.

**TABLE 1:** The radiographic OA scoring system

Score	Level	Radiographic characteristics
0	Healthy	Normal
1	Mild	Minimal periarticular new bone formation or regional joint space narrowing.
2	Moderate	Mild subchondral bone sclerosis, remodeling of the femoral head and neck appeared associated with new bone formation.
3	Marked	Pronounced coxa‐femoral subluxation, a large intra‐articular osseous body, more obvious remodeling of the femoral head and neck and acetabulum.

### *Statistical Analysis*

Statistical analysis was performed using the Statistical Package for the Social Science software (SPSS, version 22.0, IBM, New York, NY, USA), the semi‐quantitative scores were calculated and examined with independent‐sample Kruskal–Wallis H tests. For those analyses in which the overall test was significant (*P* values less than 0.05), pairwise comparisons were completed using the Mann–Whitney U tests controlling for type I error across tests with the Bonferroni adjustment and *P* values less than 0.0167 would be considered as statistically significant.

## Results

### *General Condition: Intraoperative Appearance and Postoperative Recovery*

**Fig 4:**
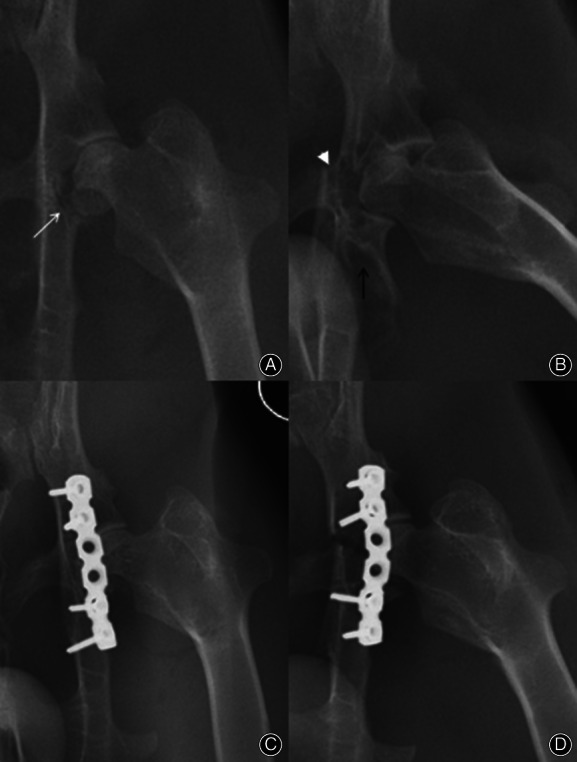
Representative radiographs before and after fixation: (A) and (C): anteroposterior (AP) radiographs of a pure dorsal wall fracture (white arrow) of acetabulum before and after ORIF; (B) and (D): anteroposterior (AP) radiographs of a transverse fracture (white arrowhead) presented with an enlarged dorsal fragment (black arrow) that involved all of the caudal part of acetabulum before and after ORIF.

Thirty‐six rabbits underwent aseptic surgeries and one animal from the ORIF group died of deep infection 4 days after surgery. Among the 24 acetabular fracture models, three were associated fractures (dorsal wall plus transverse fractures), in which one was simultaneously accompanied by dorsal dislocation and another one presented an enlarged fragment that involved all of the caudal part of acetabulum. The other 21 were pure dorsal‐wall fractures as proposed (Fig. 4), of which four were accompanied by a small cortical debris of various sizes at caudal medial corner of the fragment. All of the modeled joints were stable after reduction excepted for the one rabbit with an enlarged fragment, which also regained its stability after internal fixation.

Rabbits in the sham group favored the traumatized limb for 3–5 days after operation and the ORIF rabbits often required as long as 2 weeks to return to normal gait. However, most animals (11/12) in non‐ORIF group kept limping to some extent and restricted joint movement, in particular with extension and abduction. Two weeks after operation, all of the 35 alive rabbits were examined to check the force of the traumatized limbs and compared to the contralateral ones. There was no obvious muscle weakness when they kicked, which correlated with sciatic nerve injury.

### *Macroscopic Description*

**Fig 5:**
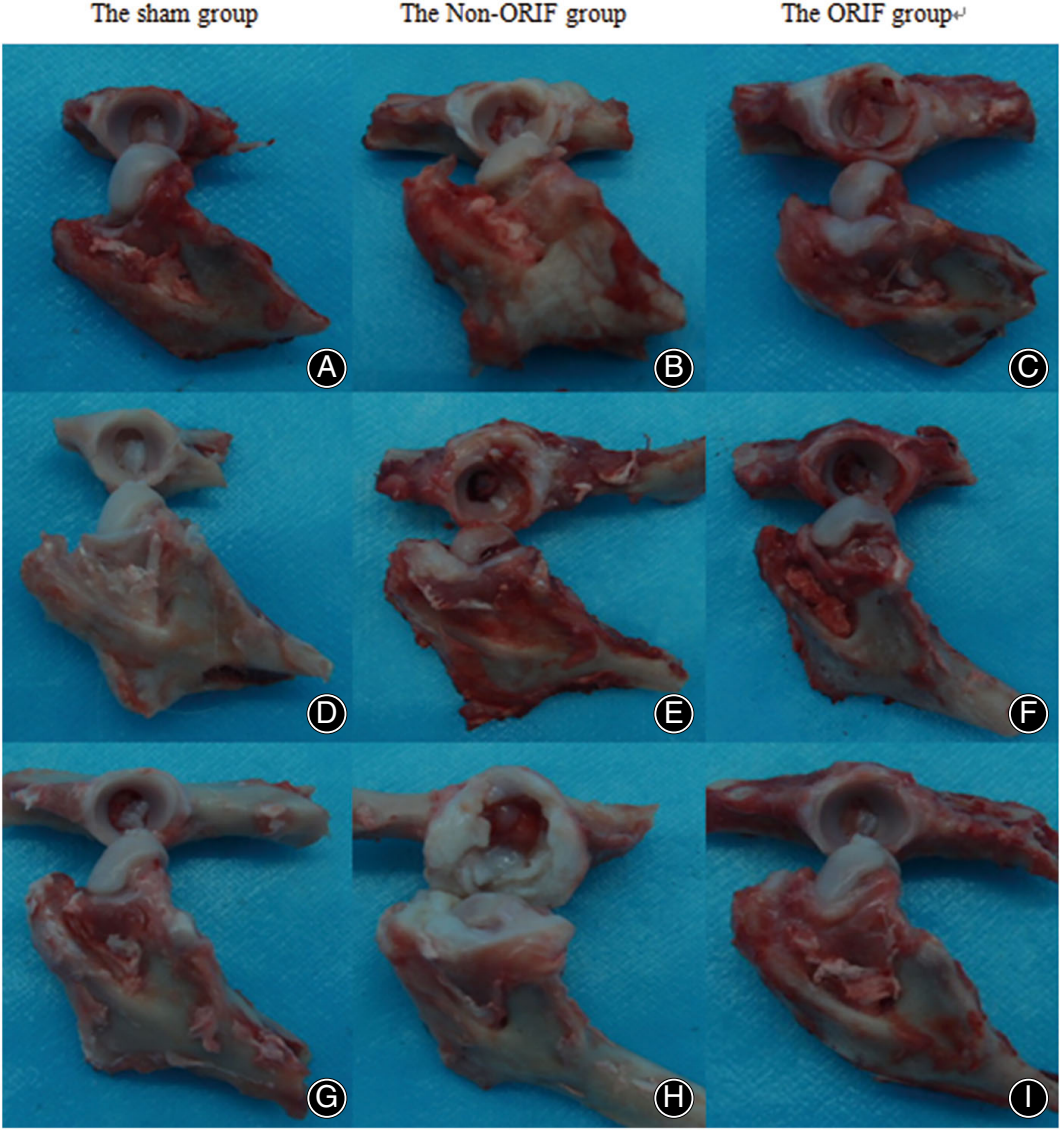
Macroscopic views of different groups at 3 weeks (upper row), 6 weeks (middle row), and 6 months (bottom row), respectively: At 3 weeks, articular capsules and glenoid labra of modeled acetabula showed mild thickening, in particular in the non‐ORIF group. The fracture lines were still distinguishable (B and C); A 2 mm step‐off was observed near the fragment of the non‐ORIF group (B); At 6 weeks, a lot of osteophytes around the acetabulum and the fragment was partially absorbed, while obvious abrasions on femoral heads were opposite the previous fracture site (E). Cartilage degeneration and structure of the joint were kept in much better condition in the ORIF group (F). At 6 months, the non‐ORIF group showed a hypertrophic hip joint and the acetabular fossa was almost impossible to identify (H). There were some cartilage defects with subchondral bone appearance in the acetabulum and the spheric femoral head was flattened and malformed. The hip in the ORIF group kept a much better structure (I).

Three weeks after surgery, articular capsules and glenoid labra of animal models showed mild thickening, in particular in the non‐ORIF group (Fig. 5). In the modeled acetabula, the fracture lines and profile of fragments were still distinguishable, the structures of the acetabula and sphericity of femoral heads remain similar to the sham group. Articular cartilages from the two modeled joints had mildly rough surface, and step‐offs between 1‐3 mm were observed near the fragments of the non‐ORIF group. In addition, the non‐ORIF group presented pale and lackluster cartilages when compared to the sham and ORIF groups.

At 6 weeks, all fractures healed and there were a lot of osteophytes around the non‐ORIF acetabula while capsules and glenoid labra further thickened. The fragments healed in displaced position and were partially absorbed. The cartilage defects were filled by what appeared to be fibrocartilage with uneven articular surface. Characteristics of cartilage degeneration were evident in the joint as well obvious abrasions located on femoral heads that were opposite to the previous fracture sites. Comparatively speaking, cartilage degeneration and structure of the joint were kept in much better condition in the ORIF group, though not as good as in the sham group.

The non‐ORIF group showed hypertrophic hip joints while capsules and glenoid labra were severely thickened and stiffened at 6 months. The structures of acetabular fossae were almost impossible to be identified and there were some cartilage defects with subchondral bone appearance at the residual fossae. Besides of the yellowish cartilage, the spheric femoral heads were flattened and malformed as a result of long‐time wearing and remodeling. On the contrary, acetabula and femoral heads of the ORIF group had no significant further changes when compared to that their condition at 6 weeks.

### *Radiographic Features and Scores*

**Fig 6:**
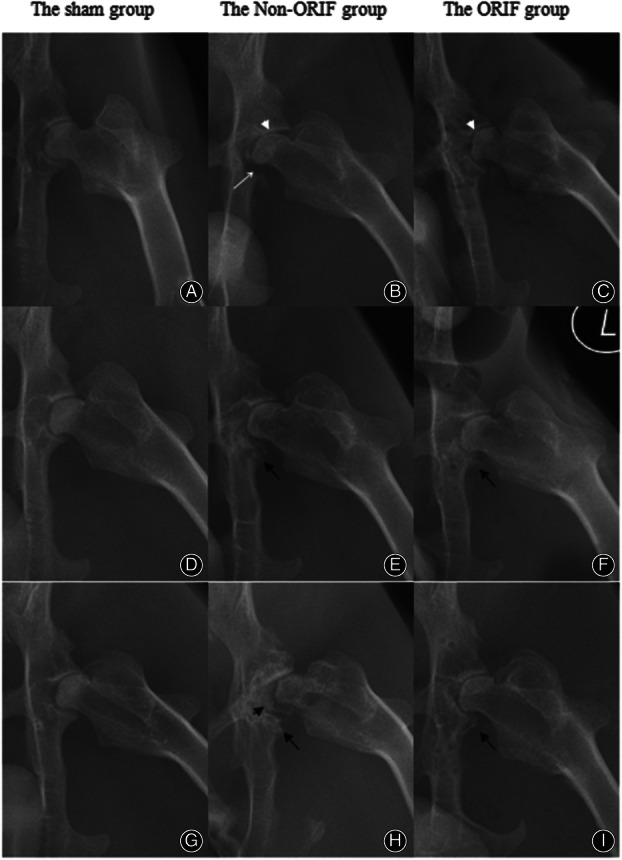
Representative radiographs of different groups at 3 weeks (upper row), 6 weeks (middle row), and 6 months (bottom row), respectively: At 3 weeks, compared to the sham group (A), there were regional joint space narrowing in the non‐ORIF and ORIF groups (B and C); one acetabulum in the non‐ORIF group showed an obvious defect near the caudal margin (white arrow); at 6 weeks, the non‐ORIF group demonstrated uneven radiodensity accompanied by lots of osteophytes growing (E), while there were limited osteophytes in the ORIF group (F). At 6 months, the non‐ORIF group showed a severely malformed acetabulum with coxa‐femoral subluxation (H), and a large osseous body occupied the joint space (black arrowhead). No more osteophytes grew in the ORIF group (I) (white arrowhead: regional joint space narrowing; black arrow: osteophytes).

As the radiographs showed (Fig. 6), there was no other characteristic PTOA change for all three groups but regional joint space narrowing in the two modeled groups (the non‐ORIF and ORIF groups) at 3 weeks. In the non‐ORIF group, one acetabulum appeared an obvious defect near the caudal margin, which was correlated with unstable and displaced fragments. All of the acetabula in the ORIF group showed intact fossae as the sham group, but there were some regional radiolucent holes, which were left by removed hadwares.

At 6 weeks, compared to the sham group, the non‐ORIF rabbits demonstrated uneven radiodensity near the previous fractured sites. Regional sclerosis was accompanied by osteophytes growing. One rabbit in this group represented mild femoral head remodeling. Other than radiolucent holes left by screws, there were limited osteophytes which were much smaller than that of non‐ORIF group at the caudal edge of acetabula in the ORIF group.

The radiographs of the non‐ORIF group showed malformed acetabula with shallow fossae and large osseous bodies occupied the joint space at 6 months. Furthermore, long‐time bearing and further joint remodeling led to flattened femoral heads with coxa‐femoral subluxation. Osteophytes expanded around the joint. Subchondral bone of acetabula and femoral heads became obviously sclerotic. Compared to the radiographs of prior intervals, the subchondral bone of acetabula in the ORIF group showed mild sclerosis at 6 months and not much more growth of osteophytes.

**Fig 7:**
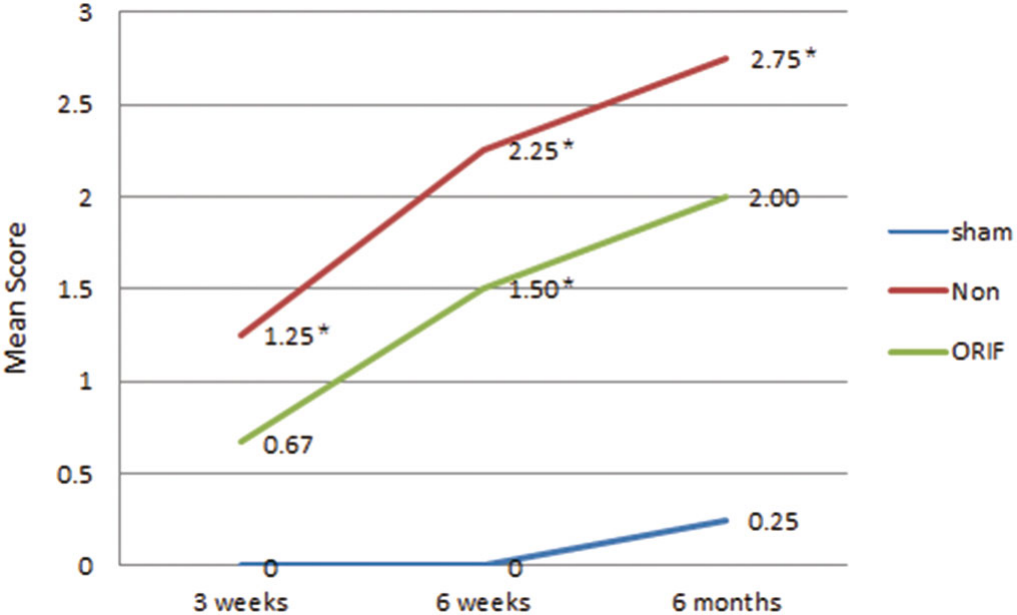
The trends and comparison of semi‐quantitative scores in different groups (^*^represented a significant difference when compared to the sham group). There were both trends toward increasing radiographic scores according to the semi‐quantitative system in the non‐ORIF and ORIF groups from 3 weeks to 6 months.

There were trends toward increasing radiographic scores according to the radiographic scoring system in the non‐ORIF and ORIF groups from 3 weeks to 6 months (Fig. 7). Three weeks after surgery, the non‐ORIF group showed significant higher scores when compared to the sham group (*P* = 0.011, <0.0167), while there was no statistical significance between the sham and ORIF groups. Although both non‐ORIF and ORIF had significantly higher scores when compared to the sham group at 6 weeks (*P* = 0.011, 0.013, respectively, <0.0167), there was no significant difference between the two modeled groups. At 6 months, one acetabulum in the sham group was given a score of 1 for minimal periarticular osteophyte that was considered as natural degeneration. At this interval, three quarters of the rabbits in the non‐ORIF group were given the most severe scores of 3 and showed statistically significant difference when compared to the sham group (*P* = 0.015, <0.0167), but no significant difference was observed between the ORIF and sham groups or the non‐ORIF and ORIF groups.

## Discussion

### *Animal Models*

Studies investigating potential pathogenesis and pathology of post‐traumatic osteoarthritis following fractures of acetabulum and evaluating the efficacy of therapeutic treatments requires subjects with hips which are large enough to apply surgical interventions. Small species such as mice, guinea pigs, and Syrian hamsters suffer from their limited joint sizes and operations are technically demanding or impossible. Although Olson *et al*. developed an *in vivo* Nubian goat model for simulating human posterior wall fracture of acetabulum, like some other large animal models, this model may be limited by its high cost, long degenerative course, restriction of experiment condition, and the difficulty in carrying out large sample studies^20^, ^24^.

Ariz *et al*. demonstrated that rabbits mimicked many characteristics of human osteoarthritis such as degenerating with aging and increased risk with weight^23^. Our previous study also presented the similarity between the hips of rabbit and humans by comparison of lateral center‐edge angle (LCEA) and acetabular‐head index (AHI), and the hips of modeled rabbit appeared to show characteristic changes of PTOA 3 months after acetabular fracture^21^. Therefore, we conducted the present study for further research on PTOA following acetabular fracture.

### *Fracture Mechanism*

As the most common fracture pattern of acetabulum, posterior wall fracture always happens in motor vehicle crashes^2^, ^4^, ^5^, ^7^. When the knee dramatically impacts with a dashboard, the force is transmitted through femoral shaft, whereafter acetabulum is fractured by the femoral head (dashboard impaction mechanism)^25^. In addition, according to the study of Judet *et al*., a posterior wall fracture happens when the hip is positioned at approximately 0° of abduction in the frontal plane and the exact location of the main fragment varies with the amount of flexion of the hip at sagittal plane^1^. So, a standard position like this was adopted during rabbit modeling to simulate a dashboard‐impacting injury.

The fracture was designed to be a trapezoid fragment in order to mimic the usual crescent‐shaped posterior wall fracture in humans as Waller *et al*. described^25^, ^26^. Moreover, when the fragment involves 25%–50% of acetabulum, the stability of the hip depends on the integrity of the joint capsule^27^. Therefore, the width of fragment was kept at less than 50% of the retroacetabular surface and capsular attachment to the fragment was also carefully preserved. All of the modeled joints were stable after reduction except for the one enlarged fragment that involved all of the caudal part of the acetabulum, which also regained its stability after internal fixation.

Besides, drilling and connecting procedures during outlining a fragment should only pass through the outer cortex of the retroacetabular surface, so that articular cartilage was kept intact and would get sufficient support from surrounding tissues. When the gravity accelerated mass impacted to the knee, blunt trauma would happen to the cartilage surface. Repo *et al*. reported an approximate 25 MPa stress would destroy the cartilage tissue^28^. Milentijevic *et al*. revealed that a stress more than 40 MPa would result in comprehensive cartilage matrix damage and full‐thickness chondrocytes death (80%–100% of chondrocytes) in an *in vivo* rabbit model^18^. Our previous study also recorded an approximate 45 MPa stress on average during impaction^21^, which is big enough to induce comprehensive cartilage damage as a high‐energy trauma.

### *Characteristics of the Rabbit Model*

In the present study, 21 out of 24 (87.5%) acetabula presented with proposed fractures and the other three were associated fractures (dorsal wall plus transverse fracture) accompanied by dorsal dislocation or not, which may be correlated with individual variation such as microstructural change and bony strength. The fracture model demonstrated a high consistency and reproducibility.

As the macroscopic and radiographic changes showed, the hips in two modeled groups revealed progressive degeneration as the time progressed when compared to the sham group. At the end interval of the study, the hips from the non‐ORIF group exhibited severely malformed and hypertrophic joints, which was consistent with the lost function of the traumatic limbs and the trend of increased radiographic scores as the semi‐quantitative system showed. These kind of degenerative changes may be the result of different biomechanical environments with changed load distribution^29^. Reduction quality and restoration of stability are the two important factors related to the therapeutic effect^30^. Letournel found higher incidence of PTOA happened in the imperfect reduction group^2^. Giannoudis *et al*. also revealed a negative relationship between PTOA and the quality of reduction^4^. The outcome of the present study also demonstrated that ORIF surgery could effectively improve the degenerative process of post‐traumatic osteoarthritis. However, besides the biomechanical influence, the biochemical mechanism of PTOA must contribute to the outcomes, however the details of this remain unclear. A consistent and reproducible animal model is needed for further investigation.

### *Limitations*

There are several limitations to the present study. First, in addition to the magnitude of load, injuries would also be affected by loading rate^31^, ^32^, ^33^, which cannot be separated in the present drop‐tower impacting configuration. Therefore, the rabbit model cannot completely simulate motor vehicle dashboard‐type injury. Secondly, although the hip joint of rabbit is similar to human hip joint to some extent, the characteristics of loading and movement of quadrupeds are obviously different; thus, conclusions from rabbit model cannot be directly translated to humans. Thirdly, the present study is just to introduce this kind of model and preliminarily describe the macroscopic and radiographic changes at different intervals. More details about pathologic changes and potential pathogenesis would be revealed soon after, which is included in the content of the next stage.

### *Conclusion*

The fracture model with high consistency and reproducibility showed progressive post‐traumatic osteoarthritic changes, which could be improved by an open reduction and internal fixation surgery. The model provided an alternative selection for investigating potential pathogenesis and pathology of post‐traumatic osteoarthritis following fracture of acetabulum.
